# RNA Extraction from Cartilage: Issues, Methods, Tips

**DOI:** 10.3390/ijms24032120

**Published:** 2023-01-20

**Authors:** Stefania Pagani, Melania Maglio, Laura Sicuro, Milena Fini, Gianluca Giavaresi, Silvia Brogini

**Affiliations:** 1 Surgical Sciences and Technologies, IRCCS Istituto Ortopedico Rizzoli, 40136 Bologna, Italy; 2 Department of Pathology, IRCCS Istituto Ortopedico Rizzoli, 40136 Bologna, Italy; 3 Direzione Scientifica, IRCCS Istituto Ortopedico Rizzoli, Via Di Barbiano, 40136 Bologna, Italy

**Keywords:** cartilage, RNA, extraction methods, fresh tissue

## Abstract

The increase in degenerative diseases involving articular cartilage has pushed research to focus on their pathogenesis and treatment, exploiting increasingly complex techniques. Gene expression analyses from tissue are representative of the in vivo situation, but the protocols to be applied to obtain a reliable analysis are not completely cleared through customs. Thus, RNA extraction from fresh samples and specifically from musculoskeletal tissue such as cartilage is still a challenging issue. The aim of the review is to provide an overview of the techniques described in the literature for RNA extraction, highlighting limits and possibilities. The research retrieved 65 papers suitable for the purposes. The results highlighted the great difficulty in comparing the different studies, both for the sources of tissue used and for the techniques employed, as well as the details about protocols. Few papers compared different RNA extraction methods or homogenization techniques; the case study reported by authors about RNA extraction from sheep cartilage has not found an analog in the literature, confirming the existence of a relevant blank on studies about RNA extraction from cartilage tissue. However, the state of the art depicted can be used as a starting point to improve and expand studies on this topic.

## 1. Introduction

Nucleic acid extraction has become a routine procedure in molecular biology, leading the way to the characterization, isolation, and manipulation of the main molecular components of cells and organisms. It is known that purification of intact RNA is the primary step of many molecular biology techniques, including PCR, but RNA is a very labile molecule extremely sensible to degradation by RNases ubiquitously present. Considering these aspects, a well-performed technique of RNA extraction able to provide a high-quality, undegraded, and abundant product is needed. In addition, the tissue from which the nucleic acid must be extracted also plays an important role in the quality of the final extraction product. In the orthopedic field, there are many technical difficulties deriving from handling connective tissues composed primarily of a tough matrix with very few cells, such as bone, cartilage, ligaments, tendons, or intervertebral discs, which are hypocellular and resistant to tissue disruption [1]. Due to these problems, most research aiming to understand physiological and/or pathological mechanisms, as well as the gene expression profiles of these tissues, is performed on 2D monolayer cell cultures or 3D in vitro cultures that use exogenous biomaterials as support [2,3]. These investigations are normally carried out on chondrocytes isolated from fresh tissue and expanded in cultures, which do not fully recapitulate the pathophysiological condition [4]. Chondrocytes in vivo are in a stationary physiological state, while in vitro, the cells are in active proliferation but exposed to the known risk of dedifferentiation. For this reason, RNA yield can be expected to be higher in chondrocyte culture than in fresh processed tissue but not fully representative of the cartilage.

It follows that the analysis of these cell types in their complete and physiological microenvironment would be appealing since it would provide a better representation of gene expression profiles in vivo [5]. Obtaining RNA directly from musculoskeletal tissues, such as cartilage, at a concentration and quality suitable for downstream analysis would be more interesting and useful in order to reveal molecular alterations that underlie various pathologies and also in the perspective of finding out possible targeted therapies. Unfortunately, this clashes with the technical limits due to the processing of such connective tissues.

In a previous work of ours, we defined and validated a method useful to extract RNA from osteoblasts starting from the fresh calcified human bone samples, focusing on obtaining a suitable RNA quality in terms of purity and integrity, as well as identifying a set of reliable reference genes [6]. The present review arises indeed from the practical need to extract RNA from fresh cartilage tissue, the handling of which is not supported by an adequate literature’s background, useful to guide the researcher to obtain a clean and sufficiently abundant RNA. Despite the similar technical difficulties involved both in bone and cartilage tissue, the contribution of the literature on the subject is evidently unbalanced toward the bone. Though bone presents undoubtable obstacles deriving from its mineralized components, studies on cartilaginous tissue are seriously hampered by problematic RNA isolation due to low cell density (only 1-to 5% of the total mass) as well as abundant and dense extracellular matrix, composed mainly of collagen, GAGs and proteoglycans [7]. In fact, these extracellular compounds are known to tend to co-precipitate with the RNA as well as to be spectrophotometrically read at the same wavelength, thus preventing a correct quantification of the nucleic acid [8,9,10]. In addition, proteoglycans are potent inhibitors of PCR [11].

Since a large variety of genes and their products are known to play a role in the pathophysiology of various osteoarticular diseases, even more it follows that a large amount of pure RNA is needed to perform all the possible investigations. The gradual rise of the age of the worldwide population is necessarily associated with the increase in several joint diseases, which OA is the most common. It involves up to 15% of the adult population [12,13], so representing an important burden of the public health system. Osteoarthritis (OA) is considered an inflammatory, chronic and degenerative disease, supported by inflammatory cytokines and catabolic mediators such as metalloproteases (MMPs) and aggrecanases. This leads to a progressive degradation of the ECM of articular cartilage until its complete erosion, partially caused also by the limited ability to regenerate [14]. In this scenario, merely reported here as paradigmatic example of joint disease, it is our belief that a molecular analysis could provide relevant information about all these elements and the pathways regulating a pathologic process. Furthermore, the progressive degeneration of the articular surfaces during OA determines a strong reduction of the cartilage portion and therefore of available tissue. This is an aggravating factor in approaching this type of sample and confirms the importance of developing a suitable method to obtain high-quality amount of RNA from cartilage. The possibility of having RNA samples from whole tissue could not only allow a better understanding of the physiological mechanisms that regulate the cartilage but it could also allow to test the effectiveness to different treatments, both physical (e.g., ultrasounds, pulsed electromagnetic fields, rehabilitation) [15,16] and pharmacological (analgesics, opioids, non-steroidal and steroidal anti-inflammatory drugs, but also glucosamine and its derivatives) [12,17].

For these reasons and based on increasing incidence of degenerative diseases involving articular cartilage fraction and the related interest on this issue, we wanted to deepen our knowledge on the techniques for extracting RNA from cartilage and assess their differences through a review of the literature whose results are described below. Finally, our personal experience was reported.

## 2. Literature Data Searching

**Figure 1:**
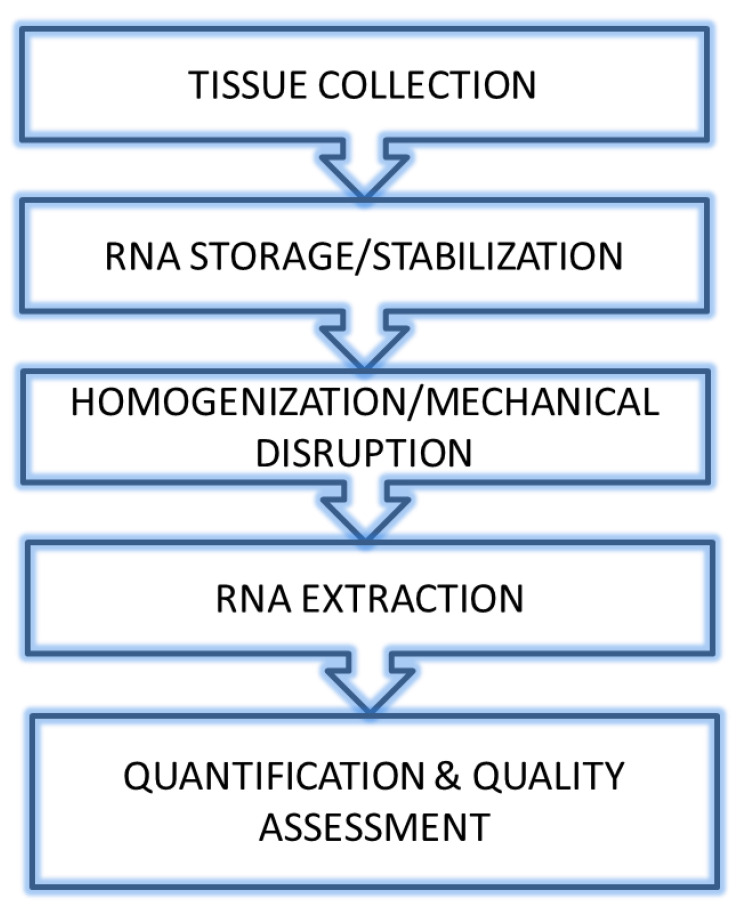
Flowchart of the main steps of RNA extraction.

DNA and RNA extraction mainly follows protocols with standardized reagents, many of which are available in commercial kits. Figure 1 shows a flowchart of the main steps involved in RNA extraction from fresh tissue to RNA ready for subsequent evaluations. Regardless of the protocol used, successful extraction of high-quality nucleic acid from biological tissues requires the disruption of the tissue and cellular structures, denaturation of nucleoprotein complexes, inactivation of nucleases, for example, ribonuclease (RNase) for RNA extraction, and nucleic acid purification.

Literature data searching was performed on 2 databases: www.pubmed.com (accessed on 1 July 2022) and www.webofknowledge.com (accessed on 1 July 2022). All publications from the start of scientific literature to June 2022 were included. Additional studies that were not found by our initial search were identified analyzing the reference lists from the included articles.

The research topic, rather specific in the technical details to be identified and rather general in its applicability at the same time, prompted the authors to perform research with a “broad” strategy. More in detail, the following key terms, and their combinations were used: “RNA AND (extraction OR isolation) AND cartilage”, “RNA AND extraction AND cartilage”) on both databases. The limits set were the English language, reviews, and other manuscript forms. Given the relative novelty represented by molecular biology, no publication date limits have been entered. Additional studies that were not found by our initial search were identified by analyzing the reference lists from the included articles. After submitting the entire list obtained to the public reference manager https://www.zotero.org (accessed on 1 July 2022) program, in order to delete duplicate papers, a careful selection of them was made on the basis of title, abstract, and, in most cases, by reading the section “Materials and Methods”. The criterion for which the selection of the articles to be included in the review was based on the study of the different cartilage extraction methods from intact tissue, including animal studies and clinical studies.

Of the 433 retrieved papers resulting from literature research carried out on PubMed and Web of Science Core Collection databases, only 15% referred to RNA extraction from mature cartilage tissue. Among them, only few papers report references on quality and purity assurance and not all specify the results related to these parameters.

## 3. Tissue Collection and Storage Condition

Tissue collection and, mostly, the subsequent storage conditions represent the first critical step for the isolation of suitable-quality RNA. For this reason, precautions are needed to be carry out to diminish RNA degradation. The most ideal tissue specimen is one that carries a complete unaltered representation of the in vivo tissue, as well as the perfect procedure is one that immediately processes the fresh samples for RNA extraction [18,19,20,21,22,23,24,25].

However, logistic problems can cause a delay in sampling, compromising the quick tissue processing for RNA isolation, making it necessary to rapidly snap-freeze the clinical samples and store them at −70/80 °C for several days [26,27,28]. Long not-controlled time ex vivo before freezing is believed to have significant effects on RNA integrity and mRNA expression levels and could consequently influence results in gene expression studies [29]. Maintaining samples at low temperature have the effect of minimizing endogenous RNases activity, which is a relevant precaution for a successful RNA extraction [13]. Even when samples cannot be immediately frozen, they are kept at a controlled, suitable temperature. For example, Hutchinson et al. affirm keeping cartilage samples in PBS on ice during dissection [20].

The majority of the studies that performed RNA extraction from fresh cartilage samples, straightaway frozen the tissue in liquid nitrogen before performing the extraction procedure immediately after [8,30,31,32,33,34,35,36,37,38,39,40,41,42,43], or stored them at −80 °C after freezing, until subsequent processing [1,7,9,10,11,22,44,45,46,47,48,49,50,51,52]. Alternatively, when immediate tissue freezing is not possible, the use of an RNA-stabilization solution should be considered. Tissue can be stored in an RNA-preservation medium that rapidly permeates tissues to stabilize and protect cellular RNA, such as RNA*later*™ solution, and stored at −4 °C [53] or −80 °C [54] minimizing the need to immediately process tissue samples. About the use of such solutions, Ruettger et al., consider it not advisable and affirm that such reagents can cause dehydration of the cartilage resulting in subsequent difficulties in the homogenization step using a microdismenbrator, except if a scalpel is used for dissection [55]. However Peeters et al. affirm that the tissue storage in an RNA*later*™ Solution, and RNA clean-up kits after isolation, did not improve the quality of the obtained RNA [11]. Differently from these storage methods, Baelde et al. snap-frozen cartilaginous tissue in CO_2_, cooled in 2-methyl-butane, and stored at −70 °C until use [56].

After sample storage at −80 °C, thawing represents another delicate but controllable step to the RNA isolation. In fact, cell membranes are disrupted by freeze-thaw cycles and this process could degrade RNA transcripts due to the intrinsic RNase activity, which is present in a variable extent in all tissue extracts. Avoiding RNA degradation represents a major challenge along the chain of RNA extraction; in vivo during surgery, ex vivo during specimen transport to the laboratory as well as during thawing and manipulation after snap-freezing [57].

Regarding this first step, literature data agree on the importance of few precautions: a quick treatment of specimens to inactivate degrading enzymes; fresh unfixed tissue is better than chemically fixed tissue; short period of storage yields better quality and quantity of nucleic acid; nucleic acid preservation is enhanced by thawing the tissue as quickly as possible [58].

### 3.1. Homogenization

The term “homogenization”, in general, describes the breakdown of tissue structure to form a homogeneous suspension or emulsion of cellular fragments through a mix of mechanical actions such as maceration or crushing or by chemicals such as detergents or organic solvents. Due to the high amount of intercellular matrix that hinders the extraction of RNA from the cells, immersed and hidden in the matrix itself, a homogenization step is essential, to pulverize the tissue.

Different approaches are available for tissue homogenization, including rotor-stator homogenization, bead-milling homogenization and grinding with a mortar and pestle. They are basically mechanical approaches in which cartilage tissue can also be disrupted in different working buffers. In order to exemplify, we decided to refer to these approaches as “mechanical” in order to distinguish them from those methods that use liquid nitrogen during the entire procedure or in a part of it, referred to as “criopulverization”.

#### 3.1.1. Mechanical Homogenization

The traditional and easier approach to pulverize from cartilage is represented by a chilled pestle and mortar [39]. Similarly, frozen cartilage was crushed with a steel cylinder in a steel tube (mortar and pestle), previously chilled in dry ice [39,41]. However, this method has several drawbacks, because it is time-consuming, especially when processing many samples and it could not be sufficiently efficient with tougher tissues such as cartilage, expecially with frozen tissue.

Several authors utilize homogenization instruments such as MagNA Lyser (Roche) [11,47,53] and Polytron [21,22,49,54,56,59,60] instruments. The first automatically disrupts cells or other biological materials by the presence of beads into special tubes: the oscillation of the instrument agitates the contents of the tubes up and down at extremely high speed with a slight twisting motion, thus disrupting nearly instantaneously the samples that collide with the beads. Polytron, on the contrary, works such as an immersion mixer and cartilage tissue is usually immersed in an extraction solution. Baelde et al. dissolved frozen material in TRIzol^®^ and homogenized it with a dispersing instrument (ULTRA-TURRAX^®^), that enables to work at high circumferential speeds even with small rotor diameters [56]. Conversely, Kwan et al. used fresh tissue, homogenized by Polytron Ultra-Turrax in 4M guanidinium isothiocyanate (GITC) containing 5 mM sodium citrate, 0.1 M β-mercaptoethanol, and 0.5% Sarkosyl [21]. Similar denaturating solutions was used by Kääpä, Robson and Re that purposed a homogenization made by Polytron for 5–7 min at 4 °C on ice [49,59,60]. Minimal changes were introduced as regard the procedures of Grumbles et al. and Leistad et al., [22,61]. Eventually, Larson et al., homogenized cartilage samples using the FastPrep-24 homogenizer that, differently from the others Polytron systems, disrupts cells through multidirectional, simultaneous beating of specialized lysing matrix beads on the sample material [54]. The detailed parameters of the mechanical homogenization techniques, when reported, and the solution utilized are shown in Table 1 and Table 2.

#### 3.1.2. Cryopulverization

Particular instruments such as a freezer mill, a microdismembrator or, simply, a frozen mortar and pestle can be used for this purpose [39]. The most frequently used system is the freezer mill, which is an impact grinder using a steel impactor moved back and forth between the metal ends of a vial containing the sample by strong magnetic fields, while the vials are immersed in liquid nitrogen [8,9,11,19,33,34,37,38,45,48,66]. A different kind of mill provides for frozen cartilage samples in a precooled stainless-steel canister with stainless-steel grinding ball and immersed in liquid nitrogen for 2 min [43]. The tissue is then desegregated using an oscillating milling machine for 2 to 3 min at 30 Hz.

The microdismembrator, similarly, is suited for fast homogenization of problematic samples, previously frozen in liquid nitrogen. It works with beads, but not in nitrogen immersion. In particular, Mikro-Dismembrator S (B. Braun Biotech International) with a maximum frequency of 50 Hz is especially well suited for fast homogenization of samples frozen in liquid nitrogen [1,10,32,35,46,46,51,62,68]. Moreover, with the same tool the frozen tissue can also be pulverized by adding denaturating solutions such as TRIzol^®^ or Tri Reagent to the samples, thus accelerating the entire process [35,51,62]. A different criopulverization method homogenizes the cartilage in a solution of 6M guanidine hydrochloride, 1% Sarkosyl, and antifoam A with a Tekmar tissue homogenizer. With this instrument cooling is guaranteed by the intermittent dipping of the tubes in liquid nitrogen [52]. Table 3 summarizes the detailed parameters of cryopulverization protocols.

## 4. Extraction

**Figure 2:**
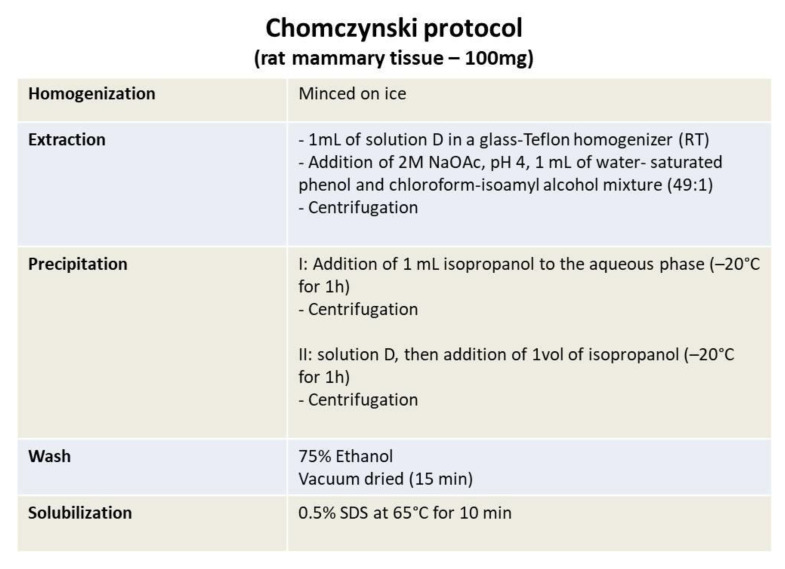
Protocol of RNA isolation by Chomczynski et al. [65].

Nowadays, a number of approaches to RNA extraction are available, which are basically a variation of the most common and oldest RNA extraction technique: the AGCP extraction, commonly referred to as the “TRIzol^®^” RNA extraction method, first described by Chomczynskj and Sacchi [65] (Figure 2). Alternatively, they are based on a solid phase approach using glass fiber filters. The method developed by Chomczynskj arises from the need to optimize the time-consuming procedure previously developed by Chirgwin et al. [69] based on GITC, one of the most effective protein denaturants able to efficiently denature endogenous ribonucleases, which is very effective but requires long hours of ultracentrifugation through a cesium chloride (CsCl) cushion. Compared to Chirgwin’s method, he reported a better ratio (A260/A280) and yield (µg RNA/mg tissue) [65]. In this way, Chomczynskj’s new method allowed the simultaneous processing of a large number of samples. Table 4 reports some examples of the principal steps of the entire extraction process focusing on the modifications to the phenol-chloroform technique performed by different authors (Table 4A) and to the methods that involve the use of salts.

The phenol-chloroform technique has many advantages due to its wide application to a variety of mammalian tissues, as well as the possibility to scale the extraction phase in order to cope with different tissue volumes. A variation of this method is the single-step extraction described by Chomczynski [70] that, using a monophasic lysis reagent, allows the simultaneous extraction of RNA, DNA and proteins (Figure 2). Such reagents are sold in different formulations under different tradenames, including TRIzol^®^ (Invitrogen), TriReagent (Sigma-Aldrich), ToTALLY RNA (Ambion), and FastPrep Pro (Q-biogene). Many authors utilize salts such as CsCl or cesium trifluoroacetate C_2_CsF_3_O_2_ o CsTFA) in order to separate RNA from DNA or proteins (Table 4B). CsCl is a salt that exhibits the ability to form a gradient, after ultracentrifugation, which allows to separate cellular components on the base of density and dimension. This means that the higher-density molecules such as RNA will collect as a pellet at the bottom of the tube [71]. On the other hand, CsTFA, due to its higher aqueous solubility, can attain a higher density than CsCl [41]. RNA remains soluble in a CsTFA gradient formed by centrifugation, instead of in precipitated form as in a CsCl salt gradient [72]. Regarding these two gradient techniques, Smale and Sasse [41] explain a RNA preparation procedure based on the use of CsTFA gradient centrifugation affirming that it improves both the yield and purity of total RNA isolated from cartilage, differently from the other methods developed by Adams et al. [8], Chomczynski and Sacchi [65] or Nemeth et al. [52]. In fact, they found these latter methods inefficient to limit proteoglycan contaminations, RNA degradation as well as to increase the RNA yield.

**Table 4:** Solutions, temperatures, and times employed in the RNA extraction procedures. (**A**) Modification of the phenol-chloroform technique; (**B**) protocols that utilize CsCl or CsTFA salts.

A					
Extraction Steps (Temp/Time)	Precipitation Solution (Temp/Time)	Pellet Digestion (Temp/Time)	Pellet Wash (Time and/or Temp)	Resuspension Solution	Ref.
TRIzol^®^ (liquid nitrogen/n.r) Chloroform (RT/3 min)	Isopropanol (−20 °C/ON)	/	70% ethanol (5 min/4 °C)	Rnase-free water	[51]
2M NaOAc/phenol/Chloroform containing isoamylalcohol (ice/15 min)	Isopropanol (−20 °C/2 hs) twice	/	96% ethanol (ON/−70 °C) 80% ethanol (20 min/4 °C) 100% ethanol	Water	[22]
AGP Acid-phenol/chloroform (four times)	Isopropanol (−20 °C/ON)	Proteinase K (55 °C/3 hs)	80% ethanol	0.1 mM EDTA	[73]
Undefined extraction solution (20 min) (twice) Fractionation after the addition of phenol/chloroform reagents	Isopropanol (Twice)	/	Ethanol	DEPC-treated Rnase-free water	[28]
(1) TRIzol^®^ (37 °C/10 min) Chloroform (37 °C/10 min) (2) TRIzol^®^ (ice) Chloroform (37 °C/10 min)	Isopropanol (RT/10 min)	/	75% Ethanol (5 min/4 °C)	Rnase-free water	[74]
TRIzol^®^ (RT/15 min) Chloroform (RT/3 min)	Isopropanol (RT/10 min)	/	75% Ethanol (twice)	Rnase-free water	[37]
Phenol and guanidine isothiocyanate (RT/30 min) Chloroform (RT/10 min)	(a) Isopropanol (−70 °C/ON) (b) 70% Ethanol + minicolumns method	/	(a) 70% Ethanol (twice)	(a), (b) Rnase-free water	[32]
TRIzol^®^ (−70 °C) Chloroform (RT/2–3 min) (twice)	Isopropanol (RT/10 min)	/	Ethanol	Rnase-free deuterium oxide	[34]
Solution D (4 °C/5–7 min) Phenol/2M NaAc/Chloroform-isoamylalcohol (49:1) (4 °C/20 min) (twice)	100% Ethanol (-20 °C/ON) (twice)	0.01% ribonuclease-free proteinase K (37 °C/15 min)	75% Ethanol (twice)	Ribonuclease-free water	[49]
Solution D Phenol/chloroform (48:1) (two times)	Isopropanol (20 °C/ON)	Proteinase K (65 °C/2 hs)	4M LiCl 70% Ethanol	DEPC-treated water	[23]
TRIzol^®^ + Chloroform (4 °C/15 min) Phenol/Chloroform/Isoamyl Alcohol (25:24:1)	Isopropanol	/	70% Ethanol 7.5 M LiCl (−20 °C/30 min)	RNase-free water	[31]
**B**					
**Extraction Steps (Temp/Time)**	**Precipitation** **Solution (Temp/Time)**	**Pellet Digestion (Temp/Time)**	**Pellet Wash (Time and/or Temp)**	**Resuspension Solution**	**Ref.**
CsCl gradient centrifugation GTC Phenol/chloroform/isoamyl alcohol (25:24:1) (once) Chloroform/isoamyl alcohol (24:1)	3 M NaOAc/100% Ethanol (−20 °C/ON)	/	/	TE	[60]
Solution D CsCl gradient centrifugation 1:1 phenol/chloroform (after digestion)	3 M NaOAc/Ethanol (−20 °C/16 hs)	Proteinase K (64 °C/1 h)	70% Ethanol	DEPC-treated water	[21]
Solution D (4 °C/ON) CsCl gradient centrifugation Solution D Phenol/chloroform/isoamyl alcohol (50:48:2) + chloroform/isoamyl alcohol (24:1)	1M AcAc/100% Ethanol (−20 °C) 2M LiCl/100% Ethanol (−20 °C)	/	n.r	n.r	[20]
Solution D CsCl gradient centrifugation Chloroform/isobutanol (4:1)	4M NaOAc (-20 °C/ON)	/	DEPC-treated water	DEPC-treated water	[52]
Solution D CsTFA gradient	Ethanol	/	DEPC-treated water	DEPC-treated water	[41]
Solution D (4 h) NaOAc Phenol Chloroform/isoamyl alcohol (24:1) (ice/15 min) Phenol/chloroform/isoamyl alcohol (25:24:1) (4 °C/20 min) Chloroform/isoamyl alcohol (24:1) CsTFA gradient	AcAc/100% Ethanol (−70 °C/30 min)	/	2M NaCl/Ethanol (−70 °C/30 min)	DEPC-treated water	[43]
Solution D CsTFA gradient 4M GTC	AcAc/Ethanol	/	n.r	TE	[75]
GIT Phenol/chloroform/isoamyl alcohol (4 °C/1 h) CsTFA gradient	3 M NaOAc/100% Ethanol (−20 °C/ON)	Proteinase K (40 °C/1 h)	RNase-free DNase + 80 U of RNasin (37 °C/30 min)	DEPC-treated water	[39]

**Abbreviations:** n.r = not reported; temp = temperature; RT = room temperature; ON = overnight; hs = hours; min = minutes; NaOAc = sodium acetate; EDTA = ethylenediamine tetraacetic acid; DEPC = diethyl pyrocarbonate; AGP = acid guanidine thiocyanate-phenol; NaCl = sodium chloride; LiCl = lithium chloride; GTC = guanidine thiocyanate; TE = Tris-EDTA; AcAc = acetic acid; CsTFA = cesium trifluoroacetate; GITC or GIT = guanidinium isothiocyanate; (1) = extraction using the liquid nitrogen-grinding method; (2) = extraction using the enzyme digestion method; (a) = Method 1; (b) = method 2.

In order to decrease proteoglycan contamination of RNA, they increased the density of the CsTFA cushion in order to determine conditions under which RNA would pellet while the majority of proteoglycans would remain suspended in the gradient, obtaining the optimal density range between 1.50 and 1.60 g/mL. Different authors include the GAGs’ solubilization step by means a LiCl wash [20,23,31,49].

### Purification Kits

Some authors referred to the use of RNeasy Kit (or column) to easily purified total RNA, often from approximately 10 to 1000 mg of cartilage tissue [9,10,30,32,45,53,54,66,76,77]. The most used kits are based on the use of silica-membrane spin columns simplifying total RNA isolation. These methods combine the stringency of guanidine isothiocyanate lysis with the speed and purity of silica-membrane purification. Moreover, some authors prefer to pursue a combination of the phenol/chloroform traditional method and the purification commercial kits [30,33,35,46,47,56,62,63,78].

## 5. Quality and Integrity Assessment

Purity and integrity of RNA are critical elements for the overall success of RNA-based analyses. Due to its high instability, fragments of RNA commonly can occur in a sample, compromising the results of downstream applications [79,80].

Different quality control methods are commonly used, even if, to date there is no real consensus on the best one [81]. Table 5 reports the different quality parameters performed by the authors with the corresponding results, when mentioned.

### 5.1. OD Measurement

Spectrophotometry technology, such as NanoDrop™, provides information on RNA quantity as well as purity (i.e., A_260_: A_280_ and A_260_: A_230_ values), based on the ability of nucleic acids to absorb UV light at a wavelength of 260 nm. Quantity and quality assessment using a UV/VIS spectrophotometer should be performed at multiple wave lengths: 240 nm (absorption of possible contaminants), 260 nm (specific for nucleic acids), 280 (specific for proteins) and 320 (absorption of possible contaminants). A major advantage of the system is the very low sample consumption of 1–2 μL, which is especially important when using precious materials such as human biopsy. A ratio of absorbance at 260 and 280 nm (A_260_: A_280_) greater than 1.8 is usually considered a suitable indicator of RNA purity [82]. However, the accuracy of this method has been questioned, with a value of 1.8 corresponding to only 40% RNA [83]. The A_260_ measurement can be compromised by the presence of genomic DNA leading to an over-estimation of the actual RNA concentration.

On the other hand, aromatic amino acids absorb light at 280 nm. The A_280_ measurement is used to estimate the presence of protein but it provides no information on residual organic contamination, whose wavelength correspond to 230 nm. To estimate nucleic acid purity, also the ratio of the absorbance contributed by the nucleic acid to the absorbance of the contaminants is calculated. Acceptable ratios for purity will vary with the downstream application. However, typical requirements for pure RNA should have A_260_: A_230_ equal to A_260_: A_280_ and >1.8 [81].

### 5.2. Gel Electrophoresis

A second check involves agarose or acrylamide gel electrophoresis in which samples are loaded and nucleic acids fragments separated based on size. The gels are stained with ethidium bromide or SYBR Green dye that bind nucleic acids but are not specific to RNA and the separated fragments can be visualized by excitation of the fluorescent dye. RNA concentration can be qualitatively measured by comparing the fluorescence intensity of the RNA bands to that of known RNA standards, typically the 28S and/or 18S rRNA [84]. The RNA is considered of high quality when the ratio of 28S: 18S bands is about 2.0 and higher [80,81]. Although less expensive, these methods require a significant amount of handling time as well as significant amounts of precious RNA and the standard of a 2.0 rRNA ratio hardly is obtained in practice, especially for RNA derived from clinical samples [85]. On the other hand, lower rRNA ratio is not necessary a sign of poor quality especially if no degradation products can be detect in the electrophoretic trace [81]. Imbeaud et al., affirms that when 28S:18S rRNA ratios were calculated from identical samples but through independent runs, a 19–24% degree of variability was observed. Thus indicating that this parameter may not be considered the gold standard for RNA integrity [81].

### 5.3. Microfluidics

As mentioned above, conventional methods are often not sensitive enough, not specific for single-stranded RNA, and susceptible to interferences from contaminants as well as subjective interpretations [83]. For these reasons, many authors use microfluidics technology (Agilent 2100 Bioanalyzer) to analyze DNA, RNA, protein, and cells using sample-specific chips and express their results using the algorithm “RNA Integrity Number” (RIN), a new tool developed to remove individual interpretation in RNA quality control, which takes the entire electrophoretic trace into account and not only the ratio of 28S and 18S rRNAs [80]. Despite the above mention methods, it requires only a very small amount of RNA sample (as low as 200 pg). The use of a size standard during electrophoresis allows the estimation of sizes of RNA bands, and the measurement appears relatively unaffected by contaminants [81]. Furthermore, in terms of routinely analyzing a large number of RNA preparations, it is by far the most convenient and objective way of assessing the quality of RNA. The resulting RIN scale range from 0 to 10, with 10 indicating the maximum RNA integrity. The software estimate not only RNA degradation but also RNA concentration by comparing peak areas of a ladder with RNA fragments of known concentration and peak areas of the unknown samples. An added advantage is the ability to sort small RNA or microRNAs. On the other hand, the disadvantage is the lack of information on sample purity as well as the irrelevance for some downstream applications such as RT- PCR. Furthermore, the generated ribosomal ratios are dependent on the used capillary-electrophoresis systems that can show differences in the generated 28S:18S ratio values, sensitivity and reproducibility [83].

**Table 5:** Quality and integrity parameters evaluated by authors and the corresponding results.

Species	Sample Details (Species; Quantity; site; Pathology)	OD Measurement	Gel Electrophoresis	Microfluidics	Results	Ref.
Human	1 gr; AC; healthy and OA	A_260_:A_280_ A_230–_A_400_	28S:18S	-	2–10 μg RNA/g	[9]
	AC; 0.25–0.3 gr; OA, RA and healthy	A_260_:A_280_	-	-	1.6 < A_260_:A_280_ < 1.8	[22]
	AC; OA	A_260_:A_280_	28S:18S	RIN	RNA yield = 0.62 μg/100 mg tissue (TRIzol^®^) RNA yield = 0.65 μg/100 mg tissue (modified protocol) ↑ RIN (5.5) of modified extraction method (6.5–8.5) with respect to traditional TRIzol^®^ (0–4.5) ↓ A_260_:A_280_ (−0.16) of modified extraction method (1.64–1.90) with respect to traditional TRIzol^®^ (1.87–2.07) ↑ A_260_:A_230_ (0.91) of modified extraction method (0.18–1.91) with respect to traditional TRIzol^®^ (0.09–0.99)	[30]
	AC; 25 mm discs 0.1 mm thickness; OA	A_260_:A_280_ A_260_:A_230_	28S:18S	RIN	RNA yield = 2.26 μg/100 mg tissue 1.92 < A_260_:A_280_ < 2.12 1.27 <A_260_:A_230_ < 2.24 1.1 < 28S:18S < 2 6 < RIN < 8.6	[31]
	AC; 3–5 mm thick	A_260_:A_280_ A_260_:A_230_	-	RIN	RNA yield = 2.33 μg/100 mg tissue RIN: 7.9 ± 0.3 A_260_:A_280_: 1.8 ± 0.11 A_260_:A_230_: 1.9 ± 0.23	[37]
	1gr; AC; healthy and OA	A_260_:A_280_ A_230–_A_400_	28S:18S	-	1.8 < A_260_:A_280_ < 1.9 No RNA degradation	[45]
	AC;	A_260_:A_280_ A_260_:A_230_	28S:18S	RIN	60 < RNA yield < 124 (μg/μL) (depending on extraction method) 1.8 < RIN < 6.2 for humans (depending on extraction method)	[55]
	CC; 250 mg	A_260_:A_280_	28S:18S	-	0.1 < RNA yield < 0.5 (μg/mg) 1.9 < A_260_:A_280_ < 2.1	[56]
	AC; 500 mg; OA	A_260_:A_280_	28S:18S	-	RNA yield = 1 μg/500 mg tissue 1.6 < A_260_:A_280_ < 2.0 Minimal RNA degradation	[67]
	AC; healthy and OA	A_260_:A_280_ A_260_:A_230_	28S:18S 5S	-	n.r	[73]
	NP; 100 mg; degeneration	A_260_:A_280_	28S:18S 5S	-	n.r	[74]
	AC; healthy and OA	A_260_:A_280_	28S:18S	-	A_260_:A_280_ = 1.8:2.0	[86]
Bovine/Calf	25 mm^2^; GP	A_260_:A_280_	-	-	n.r	[23]
	Bovine; AC; 50 mg Calf; AC; 50 mg	A_260_:A_280_ A_260_:A_230_	28S:18S	RIN	60 < RNA yield < 124 (μg/μL) (depending on extraction method) 5.4 < RIN < 6.4 for bovine (depending on extraction method); RIN = 6.1 for calf	[55]
	AC; 50 mg	A_260_:A_280_	-	-	n.r	[59]
	AC; healthy and OA	A_260_:A_280_ A_260_:A_230_	28S:18S 5S	-	n.r	[73]
Dog	1 mm fragments; AC; healthy and OA	A_260_:A_280_ A_260_:A_230_	28S:18S RR	DF RIN	↑ quality for healthy samples; RIN and RR most sensitive metrics; DF most specific metric	[32]
	AC; 1–2 gr	A_260_:A_280_	28S:18S	-	A_260_:A_280_ > 1.8	[87]
Rat	AC	A_260_:A_280_	28S:18S	RIN	1.9 < A_260_:A_280_ < 2.1 7.8 < RIN < 9	[51]
	XC; 200–800 mg	-	28S:18S	-	0.2 < RNA yield < 0.6 (μg/mg)	[52]
Mice	AC	A_260_:A_280_	28S	-	n.r	[88]
Rabbits	AC; 50 mg	A_260_:A_280_	-	-	1.7 < A_260_:A_280_ < 2.2	[1]
	AC	A_260_:A_280_	28S:18S	-	0.114 < RNA yield < 0.260 (μg/mg) 1.4 < A_260_:A_280_ < 2.0 No RNA degradation	[46]
Goat	NP, AF, AC, M	A_260_:A_280_ A_260_:A_230_	28S:18S	RIN	**AC**: 1.28 < A_260_:A_280_ < 1.94 (depending on isolation kit) 0.22 < A_260_:A_230_ < 0.67 (depending on isolation kit) 3.33 < RNA yield < 153.6 (μg/mg) (depending on isolation kit) **M**: 1.38 < A_260_:A_280_ < 1.95 (depending on isolation kit) 0.19 < A_260_:A_230_ < 0.69 (depending on isolation kit) 3.66 < RNA yield < 114.9 (μg/mg) (depending on isolation kit) **AF**: 1.24 < A_260_:A_280_ < 1.94 (depending on isolation kit) 0.12 < A_260_:A_230_ < 0.47 (depending on isolation kit) 2.16 < RNA yield < 113.2 (μg/mg) (depending on isolation kit) **NP**: 0.98 < A_260_:A_280_ < 1.67 (depending on isolation kit) 0.13 < A_260_:A_230_ < 0.8 (depending on isolation kit) 2.46 < RNA yield < 102.8 (μg/mg) (depending on isolation kit) RIN: 4.45 ± 0.57 for MagNA Lyser with respect to freezer mill	[11]
Chicken	1 gr; GP	A_260_:A_280_	28S:18S	-	Frozen GP: A_260_:A_280_ = 1.79; RNA yield = 110 μg; denaturated Fresh GP: A_260_:A_280_ = 2.04; RNA yield = 98 μg; partially denaturated	[43]
Porcine	ID; healthy and injured ID	A_260_:A_280_	28S:18S	-	↑ Rna yield in the outer annulus: 8 < RNA yield < 15 (μg/100 mg) in the control discs 22 < RNA yield < 59 (μg/100 mg) in the injured discs	[49]

**Abbreviations:** n.r = not reported; AC = articular cartilage; OA = osteoarthritis; A_260_:A_280_ = 260 to 280 nm absorbance ratio; A_230_–A_400_ = 230 to 400 absorbance spectrum; A_260_:A_230_ = 260 to 230 nm absorbance ratio; NP = nucleus pulposus; DF = degradation factor; RIN = RNA integrity index; RR = ribosomal peak ratio; ↑ = higher/better; RA = rheumatoid arthritis; CC = cartilago costalis; XC = xiphoid cartilage; AF = annulus fibrosus; M = meniscus; GP = growth plate; ID = intervertebral disc.

## 6. Case Study

The research lines of our laboratory mainly involve studies on bone and cartilage. The need to be able to refer on a reliable method to extract RNA from cartilage has long been paramount, but the difficulties encountered in finding useful methods prompted us to review the literature and, at the same time, to test a technical approach.

Our approach involved pulverization of frozen sheep articular cartilage samples (n = 5), followed by five different extraction methods. Specifically, all the samples were removed with a scalpel from the femur head, weighed (average weight: 139.2 mg), and immersed in RNA*later*™ in a volume equivalent to approximately 10 times the volume of the sample. After keeping them at 4 °C ON, the samples were maintained at −80 °C until the RNA extraction. To proceed with the subsequent steps, the RNA*later*™ was carefully removed as much as possible, and the cartilage fragments were placed into the cylinder of FreezerMill (FreezerMill 6770, Spex Sample PREP, Metuchen, NJ, USA), equipped with a stainless impactor. After 10 min of precooling in liquid nitrogen, pulverization was carried out by three cycles at the impact frequency of 15 cps (2 min each cycle) and a cooling step of 2 min between the cycles. All tested extraction methods involved resuspending the sample in TRIzol^®^ reagent immediately after cryopulveritazion of the tissue and then incubated overnight at 4 °C. The subsequent extraction step was performed by the addition of chloroform in a volume equal to one-fifth of the TRIzol^®^. To obtain a cleaner product, the mixture of sample- TRIzol^®^ was distributed in several conical tubes, avoiding exceeding 1 mL for each tube, before the addition of chloroform.

In order to isolate RNA from its dense PG-rich ECM, salts such as NaCl and NaOAc, in addition to glycogen, were used in three out of five methods (1, 2, 4), with some little modifications between them.

- **Method N.1**: The aqueous phase harvested after centrifugation at 12,000 rpm (at 4 °C for 15′) was then added to 20 μg/μL glycogen, 1.2 M sodium chloride (NaCl), and 0.8 M sodium acetate (NaOAc). RNA precipitation was performed by the addition of isopropanol, 10′ of incubation at RT, and 30′ of centrifugation at 12,000 rpm at 4 °C. Finally, the obtained RNA was twice washed with 75% cold ethanol and dried under the hood.
- **Method N.2:** Method 2 is identical to 1, apart from the addition of a second TRIzol^®^/chloroform extraction step immediately after the collection of the aqueous phase. More in detail, a volume of TRIzol^®^ equal to twice the harvested aqueous phase was added. After 15′ of incubation at RT, the procedure continued with the addition of chloroform as in Method 1;
- **Method N.3:** Similar to Method 2, it included twice the passage of extraction by TRIzol^®^, but the second aqueous phase harvested was added to glycogen and a double volume of isopropanol. Different from other methods, it followed an incubation at −20 °C overnight followed by RNA extraction similar to what was described in Method N.1 but with only one wash in 75% Ethanol;
- **Method N.4:** This method, also known as “clean up”, is characterized by further steps at the end of Method 3, aimed at a better purification of the extract. The RNA obtained by Method 3 was added to a mixture consisting of glycogen, NaOAc, and isopropanol in deionized H_2_O (RNasi, DNasi free), then incubated 60′ at −80 °C and centrifuged. After washing with 75% ethanol, the sample was dried and resuspended in H_2_O. Finally, 10′ at 60 °C should increase the RNA resuspension;
- **Method N.5:** It represents the combination between the TRIzol^®^ method and the one based on the use of columns. More precisely, the aqueous phase obtained by TRIzol^®^/chloroform was added with glycogen and 75% ethanol in equal volume, then loaded on a commercial mini spin column (Qiagen RNeasy Mini Kit).

Isopropanol was used as a precipitation solution in all the methods except for Method 5, where purification was carried out on mini spin column chromatography using the manufacturer’s instructions (Qiagen RNeasy Mini Kit). In all the methods tested, 75% Ethanol was used to wash the pellet and RNase-free water to resuspend the samples.

Table 6 shows the concentration and purity of RNA isolated from cartilage using the five different methods as indicated. Spectrophotometry (NanoDrop™) technology provided information on RNA quantity as well as purity (i.e., A_260_:A_280_ and A_260_:A_230_ values). It shows that the A_260_:A_280_ values for each sample were all approximately 2 (average of 1.78), regardless of the method used, with the best result obtained for method 2. However, great variability was seen in A_260_:A_230_ values. The lowest A_260_:A_230_ values were found in the RNA samples obtained using methods 1 and 5, even if it does not correspond to the lowest concentration’s values. The best RNA values per sample’s weight were obtained using methods 1, 3, and 4. It is worth noting that higher yields were obtained by applying methods 1 and 4, which, differently from Method 3, implied salts use, suggesting their key role in RNA extraction. Regarding Method 2, a better A_260_:A_280_ value resulted in a lower RNA yield. In our experience, Method 5 seems to be not suitable.

## 7. Discussion

Despite the experience of our laboratory in the extraction of RNA from many musculoskeletal cells, the present review arose from the practical need to extract RNA from fresh cartilaginous tissue. This need is owned by insufficient knowledge in the literature due to the complexity of handling such connective tissues, which are both hypocellular and resistant to tissue disruption.

In this regard, an important issue is the use of specialized equipment such as microdismembrator or freezer mills to process the fresh cartilaginous tissue. However, such instruments could not always be available in all laboratories. Furthermore, these methods typically use columns for RNA extraction and purification, obtaining suitable RNA quality but poor yield [30]. Even when one of these tools is available, it is difficult to understand what the best parameters are to apply, due to the scarcity of information available and the differences between authors, making it difficult also to compare them. Very few articles evaluate different homogenization methods combined with different RNA isolation protocols. Ruettger et al. [55] tested alternative combinations of homogenization methods with different RNA isolation protocols in order to link the isolation of high-quality RNA to different homogenization methods. In particular, the authors used a scalpel, microdismembrator, and rotator-stator as variants for homogenizations combined differently with TRIzol^®^, RNeasy, TRIzol^®^/RNeasy, and RNAqueous extraction methods. Concerning the methods of choice for cartilage homogenization, they concluded that the methods should be chosen since the subsequent isolation approach will be used. They argued that milling associated with TRIzol^®^ reagent and RNeasy resulted in significant RNA degradation and, consequently, in false positive results in gene expression. Conversely, the milling did not negatively influence RNA yield and quality. Similarly, Adams et al. [87] compared five methods of tissue homogenization: frozen cartilage chopped with a scalpel, smashed in a stainless-steel bin under liquid nitrogen, homogenization in a GIT mixture (guanidine isothiocyanate-mercaptoethanol mixture) using a Polytron, slicing the tissue into 20 µm sections using a cryostat, and grounding under liquid nitrogen with mortar and pestle. The authors claimed some difficulties in chopping frozen cartilage into small pieces, and for this reason, they did not pursue this method further after assessing that the amount of mRNA was almost absent. Regarding Polytron homogenization, it left large chunks of tissue in the GIT solution. Among the above-mentioned techniques, the authors identified the cryostat-sliced tissues as the better in terms of RNA yield. Geyer et al. [67] have identified the microdismembrator method as the best after comparing it with other three tissue-disruption strategies, such as mortar and pestle, stator-roto homogenizer (Polytron), and freezer mill. Gan et al. [74] instead proposed two methods: the first described samples were placed in a precooled mortar, and liquid nitrogen was added three or four times to condense the cartilage and ground it into a powder using a pestle. The second method used fresh samples cut into pieces and underwent enzymatic digestion. He concluded that the enzymatic method was widely better than the other one. Ali et al. [30] proposed and compared the traditional TRIzol^®^ protocol performed on snap-frozen samples crushed with mortar and pestle and homogenized by Polytron sonication with a modified version. In particular, obtained human articular cartilage samples were firstly minced with a scalpel and incubated into culture media. Then, chondrocytes were isolated from the surrounding proteoglycan-rich matrix by a chemical digestion with trypsin in rotation with 5.0 mm glass beads, followed by an incubation in collagenase and then lysis in TRIzol^®^ [30]. Regarding this, however, Thorp et al. stressed the fact that digesting the tissue with collagenases would alter mRNA expression, thus underling the necessity to use a method of extracting RNA from intact cartilage [43]. For this reason, they compared two methods of extraction on fresh or frozen cartilage. Frozen samples were obtained by snap-freezing in liquid nitrogen, then pulverized in a freezer mill, or by crushing the frozen samples between two pieces of metal to pulverize them and by adding the powder to a denaturating solution in a Polytron. Conversely, the fresh cartilage was immersed in a denaturating solution in a Polytron instrument. The authors discussed that the paramagnetic beads method was efficient and easy to perform, especially for small pieces of tissue, allowing to obtain enough mRNA for the PCR reactions. Furthermore, using magnetic beads allowed the separation of mRNA from other constituents of the cartilage, including collagens and proteoglycans. Finally, Peeters treated four different tissues, comparing two homogenization methods: freezer mill and MagNA Lyser, concluding that for cartilage, the better results are attributable to MagNA Lyser [11]. Again regarding the quantity of RNA obtained, both the species of origin of the sample and the quality of the sample itself must be taken into account. Regarding the first point, no one of the retrieved papers that report integrity and purity outcomes, performed analysis on sheep articular cartilage, as instead we have described in our experience, so making difficult a comparison. Moreover, cartilage from animals such as cows or calves is more cellularized than human cartilage [55]. These differences result in differences both in the yield and in the quality of RNA extracts. Furthermore, it is also very rare to be able to conduct research on healthy human cartilage for obvious reasons of tissue availability. In fact, the research carried out on human samples often processes pieces of tissue having a reduced thickness because they are characterized by pathologies such as OA. This inevitably creates problems isolating RNA from a scarce sample.

Integrity and purity of RNA is an essential critical prerequisite for gene expression analysis, especially if diagnostic, therapeutic, or prognostic conclusions will be drawn, as degraded RNA represents a limit for obtaining meaningful and realistic results [89]. It is necessary to note that very few works are focused on the comparison of RNA extraction methods; that is, there are few methodological works that are also dated in time. Most of the information was drawn from experimental works that reported the chosen technique in the Materials and Methods paragraph. Nevertheless, these papers obtain their conclusions on the reliability of these methods, mainly discussing the downstream gene expression results without mentioning the difficulties encountered or discussing the results related to the quality parameters cited. However, the accuracy of gene expression evaluation is recognized to be influenced by the quantity and quality of starting RNA. Different parameters are used to assess RNA quality and integrity, all with some drawbacks.

It is worth noting that the A_260_: A_230_ does not always predict success in downstream applications. This is due to the fact that A_230_ is often constant for nucleic acid purified using a specific kit, while the amount of RNA can vary, resulting in a decreased A_260_: A_230_ ratio. Some disadvantages of using absorbance are the lack of specificity and sensitivity. In fact, the method is not capable of distinguishing between dsDNA, RNA, or ssDNA, so the amount of genomic DNA present in an RNA preparation cannot be determined by absorbance. Furthermore, contaminants that absorb around 260 nm can contribute to the final absorbance value, overestimating the nucleic acid concentration. Finally, since single nucleotides can also contribute to the reading, changes in RNA integrity are not reflected in the measurement [81]. Furthermore, considering the cartilage tissue, it is worth noting that RNA isolated from articular cartilage requires some compromise, and it is usually not as abundant as that obtained from cells [45]. In addition, the A_260_: A_280_ ratio of RNA from tissue is generally lower than that isolated from cells (proximally 1.6-1.8) due to the presence of “contaminating” extracellular matrix components [9]. To obtain RNA with a higher A_260:_ A_280_ ratio, it is also important to remove any insoluble materials. For this reason, describing his preferred method, Adams et al. suggest centrifuging and precipitating the RNA suspension with acetic acid and ethanol after CsTFA centrifugation [8]. In addition, he points out that the RNA solution on which the OD ratio is measured should be free of both GITC and CsTFA because both of them possess a specific A_260:_ A_280_ ratio that can affect the final measurement.

Regarding our A_260:_ A_280_ results, in our experience, we obtained an average value of 1.78. This value is lower than the optimal value of 2 for this parameter, but it is in line with what was reported by Aigner et al., and it could be due to the presence of extracellular matrix components, which act as contaminants. Furthermore, it does not differ much from the values reported by other authors (Table 5).

Also, with regard to gel electrophoresis evaluation, it is often reported as a further assessment of RNA integrity. Up to now, it is still questionable if we can use the 28S/18S ratio or the RIN, both based on the quantity and quality check of the ribosomal sub-units, to make a definite statement on the mRNA quality, which is the final target in qRT-PCR. Based on structural differences alone, it might be expected that the stability of mRNA differs from rRNA. Santiago et al. affirm that mRNA integrity corresponds more closely to 28S than to 18S integrity [90], while Miller et al. expected that the 18S integrity correlated better with the mRNA, as its length is more similar to that of mRNA [91] and with a less statistical chance of cleavage respect to the 28S. The competency to quickly assess RNA quality using minor amounts has become increasingly important as the following measures of mRNA transcripts have become more expensive and more comprehensive. Flage et al. affirm the necessity to look for sensitive methods comparable to an intelligent algorithm, which prove the real mRNA integrity to have a reliable answer on mRNA quantity and quality.

## 8. Conclusions

In conclusion, this paper does not claim to find an ideal method for extracting RNA from cartilage tissue, but it provides an overview of studies that have treated the topic. From these studies, only in a small part dedicated exclusively to the methodological investigation, the researcher can draw ideas for the experimentation that must be carried out.

If the complexity of the topic, still too little debated, does not allow clear and definitive conclusions to be drawn, it is still useful to have available the papers that deal with it and the considerations that arise from it.

## Figures and Tables

**Table 1:** Mechanical homogenization protocols; related parameters and used solutions.

Mechanical					
MagNA Lyser					
Number of Cycles	Time per Cycle/Time	Frequency	Cooling between Cycles (Time/Temp)		References
2	20 s	6500 rpm	1 step (2 min/4 °C)		[53]
n.s	n.s	5000 rpm	n.s		[47]
4	40 s	6500 rpm	2 min/4 °C		[11]
Polytron and similar					
Solutions	Number of Cycles	Time per Cycle/Time	Frequency	Cooling between Cycles (Time/Temp)	References
TRIzol^®^	n.s	1 min	Ultra-turrax (3000–25,000 rpm)	n.s	[56]
GITC	n.s	5 min	1000 g	n.s	[21]
Solution D	n.s	5–7 min	n.s	4°C	[49]
Solution D	n.s	5–7 min	n.s	ice-cold	[60]
Solution D	n.s	5–7 min	speed setting: 4	ice-cold	[59]
Solution D	n.s	10 min	25,000 rpm	n.s	[22]
QIAzol lysis buffer	10	15 s	n.s	9 steps (2 min/on ice)	[54]

**Abbreviations:** n.s = not specified; GITC = guanidinium isothiocyanate.

**Table 2:** Composition and function of the solutions employed in the homogenization process.

Solution	Composition	Function	References
TRIzol^®^ (Thermo Scientific)	Monophasic solution of phenol and GITC.	It maintains the integrity of the RNA due to highly effective inhibition of RNase activity while disrupting cells and dissolving cell components during sample homogenization	[1,33,34,51,56,62,63]
RNAzol™ (Tel-Test, Friendswood, TX, USA)	Monophasic solution of phenol and GITC.	This product, a mixture of guanidine thiocyanate and phenol in a monophase solution, effectively dissolves DNA, RNA, and protein on homogenization or lysis of tissue sample	[64]
TRI Reagent^®^ (Sigma-Aldric)	Monophasic solution of phenol and GITC.	Complete and ready-to-use reagent for the isolation of total RNA or the simultaneous isolation of RNA, DNA, and proteins. It combines phenol and guanidine thiocyanate in a monophase solution to facilitate the immediate and most effective inhibition of RNase activity	[18]
Solution D	4 M GTC, 25 mM sodium citrate, pH 7; 0.5% sarcosyl, 0.1 M 2-mercaptoethanol 4 M of GITC, 25 mM of sodium citrate, pH 7.0, 0.1 M of 2-mercaptoethanol, and 0.5% N-lauroylsarcosine. 4 M GTC, 25 mM sodium citrate, pH 7; 0.5% sodium dodecyl sarcosine, 0.1 M 2-mercaptoethanol 25 mM sodium citrate, pH 7.0, containing 4 M GTC, 0.2% sarcosyl, and 0.2 M 8-mercaptoethanol	Denaturant solution	[8,22,23,60,65]
GITC	Guanidinium isothiocyanate	Chaotropic agent. Strong protein denaturant and suppressor of ribonucleases	[7]
GuCl	Guanidinium hydrochloride	Chaotropic agent used to solubilize cartilage matrix proteins and proteoglycans while simultaneously inhibiting lytic enzymes	[7,8,48,63,66,67]

**Abbreviations:** GTC = guanidinium thiocyanate; GITC = guanidinium isothiocyanate; GuCl = guanidinium hydrochloride.

**Table 3:** Cryopulverization protocols and related parameters.

Cryopulverization							
	Precooling Time	Number of Cycles	Time	Impact Frequency	Cooling Steps Between Cycles (Time)	Cool Down	References
Freezer Mill	n.s	2	1 min	15 Hz	1 step (2 min)	n.s	[66]
	2 min	5	2 min	10 Hz	4 steps (2 min)	n.s	[9]
	n.s	4	3 min	15 cps		2 min	[37]
	2 min	5	2 min	10 Hz	1 step (2 min)	n.s	[45]
	n.s		10 s–1.5 min	max	n.s	n.s	[8]
Dismembrator	/	2	2 min	15 Hz	/	/	[51]
	/		2 min	2000 osc/min	/	/	[32]
	/	2	1 min	2200 rpm	/	/	[10]
	/	n.s	2 min	2000 rpm	/	/	[68]
	/	n.s	1.5 min	2000 rpm	/	/	[35]
Other	2 min	n.s	2–3 min	30 Hz	n.s	n.s	[38]

**Abbreviations:** n.s = not specified; cps = cycles per second; osc/min = oscillations per minute.

**Table 6:** Weight, concentration, purity, and integrity of RNA isolated from sheep articular cartilage using different methods.

Extraction Method	Sheep Articular Cartilage	Weight (mg)	A_260_	A_280_	A_260_:A_280_	A_260_:A_230_	Total RNA (ng)	Ratio ng RNA/mg Sample
1	AC 1	112	9.341	5.486	1.70	0.36	10460.8	180.13
2	AC 2	114	7.780	3.966	1.96	0.67	8713.6	81.89
3	AC 3	120	13.645	7.720	1.77	0.65	15282.4	136.45
4	AC 4	120	19.623	10.932	1.80	0.90	21977.2	196.23
5	AC 5	230	0.634	0.377	1.68	0.42	711.2	3.31

Abbreviations: AC = articular cartilage.

## Data Availability

Not applicable except for the case study reported for which data are available under request.

## Acronyms and Abbreviations

DNA	deoxyribonucleic acid
RNA	ribonucleic acid
PCR	polymerase chain reaction
mRNAs	messenger-RNAs
RNases	ribonucleases
OA	osteoarthritis
GAGs	glycosaminoglycans
GITC	guanidinium thiocyanate
CsCl	cesium chloride
AGCP	acid guanidium-phenol-chloroform
RT	room temperature
NaOAc	sodium acetate
SDS	sodium dodecyl sulfate
CsTFA	cesium trifluoroacetate
LiCl	lithium chloride
dsDNA	double-strand DNA
ssDNA	single-strand DNA
rRNA	ribosomal RNA
RT-PCR	real-time PCR
ON	overnight
PG	proteoglycan
ECM	extracellular matrix
